# Successful full-length genomic cloning and characterization of site-specific nick structures of Phytophthora endornaviruses 2 and 3 in yeast, *Saccharomyces cerevisiae*

**DOI:** 10.3389/fmicb.2023.1243068

**Published:** 2023-09-12

**Authors:** Kohei Sakuta, Keiko Uchida, Toshiyuki Fukuhara, Ken Komatsu, Ryo Okada, Hiromitsu Moriyama

**Affiliations:** ^1^Laboratory of Molecular and Cellular Biology, Graduate School of Agriculture, Tokyo University of Agriculture and Technology, Fuchu, Japan; ^2^Laboratory of Plant Pathology, Graduate School of Agriculture, Tokyo University of Agriculture and Technology, Fuchu, Japan; ^3^Horticultural Research Institute, Agricultural Center, Kasama, Ibaraki, Japan

**Keywords:** endornavirus, Phytophthora, site-specific nick, northern blot hybridization, strand-specific RNA probes, high molecular weight dsRNA, homologous recombination, yeast heterologous expression

## Abstract

Two endornaviruses, Phytophthora endornavirus 2 (PEV2) and Phytophthora endornavirus 3 (PEV3), have been discovered in pathogens targeting asparagus. In this study, we analyzed the nick structure in the RNA genomes of PEV2 and PEV3 in the host oomycetes. Northern blot hybridization using positive and negative strand-specific RNA probes targeting the 5′ and 3′ regions of PEV2 and PEV3 RNA genomes revealed approximately 1.0 kilobase (kb) RNA fragments located in the 5′ regions of the two genomes. 3’ RACE analysis determined that the size of the RNA fragments were 958 nucleotides (nt) for PEV2 and 968 nt for PEV3. We have successfully constructed full-length cDNA clones of the entire RNA genomes of PEV2 and PEV3 using a homologous recombination system in the yeast, *Saccharomyces cerevisiae*. These full-length cDNA sequences were ligated downstream of a constitutive expression promoter (*TDH3*) or a galactose-inducing promoter (*GAL1*) in the shuttle vector to enable the production of the full-length RNA transcripts of PEV2 and PEV3 in yeast cells. Interestingly, a 1.0 kb RNA fragment from the PEV3 positive-strand transcript was also detected with a 5′-region RNA probe, indicating that site-specific cleavage also occurred in yeast cells. Further, when PEV2 or PEV3 mRNA was overexpressed under the *GAL1* promoter, yeast cell growth was suppressed. A fusion protein combining EGFP to the N-terminus of the full-length PEV2 ORF or C-terminus of the full-length PEV3 ORF was expressed, and allowed PEV2 and PEV3 ORFs to be successfully visualized in yeast cells. Expression of the fusion protein also revealed presence of heterogeneous bodies in the cells.

## 1. Introduction

Endornaviruses, found in plants, fungi, and oomycetes, have linear positive-sense, single-stranded RNA (ssRNA(+)) genomes of 9.8–17.6 kilobases (kb) in length. These genomes contain one open reading frame (ORF), which encodes a single polyprotein consisting of 3,217–5,825 amino acid (aa) residues with several conserved domains, such as RNA-dependent RNA polymerase (RdRP), helicase (Hel), and UDP-glucosyl transferase (UGT) (Valverde et al., 2019). Although endornaviruses have not been reported to possess capsid proteins, they have been suggested to be associated with vesicular membranes in the cytoplasm (Lefebvre et al., 1990; Moriyama et al., 1996; Horiuchi et al., 2003; Otulak-Kozieł et al., 2020).

Family *Endornaviridae* is classified into two genera, *Alphaendornavirus* and *Betaendornavirus*, based on genome size, host type, and unique domains (Adams et al., 2017). Alphaendornaviruses have been identified in plants, fungi, and oomycetes, while betaendornaviruses are only found in fungi. A common genomic structure consisting of conserved poly (C) 8–12 sequences at the end of the 3’ UTR can be found in both genera (Valverde et al., 2019). A characteristic genomic structure of alphaendornaviruses is a site-specific nick in the positive strand, while the absence of the nick structures in the minus genomic strands, and the nick is considered to occur in the intermediate double-stranded RNA (dsRNA). The nick was first discovered in *Oryza sativa* endornavirus 1 (OsEV1), which intrinsically infects Japanese cultivated rice (Fukuhara et al., 1995). It was then found in *Vicia faba* endornavirus (VfEV), which causes cytoplasmic male sterility in broad bean (Pfeiffer et al., 1993; Pfeiffer, 1998), and in *Oryza rufipogon* endornavirus (OrEV), which intrinsically infects weedy rice (Moriyama et al., 1999). Subsequently, the nicks have also been found in bell pepper endornavirus (BPEV) (Okada et al., 2011), *Phaseolus vulgaris* endornaviruses 1 and 2 (PvEV1 and PvEV2) (Okada et al., 2013), *Basella alba* endornaviruses E and R (BaEV-E and BaEV-R) (Okada et al., 2014), winged bean endornavirus 1 (WBEV-1) (Okada et al., 2017), and *Fagopyrum esculentum* endornavirus 1 (FaEV1) (Okada et al., 2021).

In most cases, endornavirus infection does not confer obvious disease symptoms in host plants, such as in CmEV-infected melon (Sabanadzovic et al., 2016), PvEV1- and PvEV2-infected bean (Wakarchuk and Hamilton, 1985, 1990; Mackenzie et al., 1988; Okada et al., 2013), OsEV1-infected rice (Moriyama et al., 1996), and BPEV-infected bell pepper (Valverde et al., 1990). In contrast, VfEV-infected broad bean causes cytoplasmic male sterility (Grill and Garger, 1981; Turpen et al., 1988), HmEV infection in the violet root rot fungus, *Helicobasidium mompa*, reduces pathogenicity of the host fungus (Osaki et al., 2006), and Phytophthora endornaviruses 2 and 3 (PEV2 and PEV3) suppress mycelial growth of the host *Phytophthora* rot pathogen (Uchida et al., 2021).

Recently, an increasing number of viruses detected in the genus *Phytophthora* have been reported, including the double-stranded (ds) RNA viruses, *Totiviridae*, *Megabirnaviridae*, and the proposed “*Fusagraviridae*” and “*Ustiviridae*,” as well as the ssRNA(+) viruses, *Narnaviridae* and *Endornaviridae*, and the negative-sense ssRNA (ssRNA(−)) viruses that are related to *Bunyaviridae* (Kozlakidis et al., 2010; Raco et al., 2022; Xu et al., 2022). In particular, endornaviruses have been detected in many *Phytophthora* species, such as *P. cactorum* (Poimala et al., 2021), *P. castaneae* (Raco et al., 2022), *Phytophthora* taxon douglas fir (Hacker et al., 2005), and *Phytophthora* rot pathogen of asparagus (Uchida et al., 2021). However, there is little information on the pathogenic mechanisms of these viral infections on the hosts. Several of these virus-infected *Phytophthora* spp. were affected in terms of growth and developmental differentiation. PEV2 and PEV3 co-infection in *Phytophthora* pathogen of asparagus promoted their zoosporangium formation, suppressed mycelial growth, and modulated specific sensitivities against fungicides (Uchida et al., 2021). *Phytophthora infestans* RNA virus 2 (PiRV-2), which is a novel unclassified virus, also promoted zoosporangium formation in *P. infestans* (Cai et al., 2019). Co-infection with Phytophthora cacutorum bunyaviruses 1 and 2 reduces the growth rates of host fungi in apple blight tissues or *in vitro* malt extract agar (MEA) medium (Poimala et al., 2022).

Although cDNA cloning is often used to study the function of genetic information possessed by RNA viruses, the construction of full-length cDNA clones is difficult for RNA viruses with large genomes. For example, full-length cDNA cloning of plant viruses in the genus *Potyvirus*, which has a large RNA genome of approximately 10 kb, has been difficult due to the prokaryotic promoter-like elements in its genome that are toxic to *Escherichia coli*, resulting in frequent mutations and deletions (Jakab et al., 1997; Ali et al., 2011). To circumvent this problem, the yeast *Saccharomyces cerevisiae* can be used because of its high homologous recombination (HR) efficiency. A seamless cloning technique utilizing HR is effective, in which multiple DNA fragments with short homologous sequences (40–50 nt) at both ends are simultaneously transformed into a linearized plasmid. It has been applied to genome cloning technologies for complex higher eukaryotes as well as simple genomic structures such as viruses (Kouprina and Larionov, 2016).

In this study, we revealed for the first time that PEV2 and PEV3 genomes exist as ssRNA(+) by northern blot hybridization and harbor nick structures in dsRNA intermediates. Further, we successfully constructed full-length cDNA clones of PEV2 and PEV3 using the yeast HR system and produced their transcripts under the constitutive expression promoter (*TDH3*) or a galactose-inducing promoter (*GAL1*). Heterologous expression of PEV2- and PEV3-derived RNA in yeast cells significantly suppressed the growth of yeast cells, which reproduces the growth suppression phenomenon caused by PEV2 and PEV3 infection in their natural host *Phytophthora* spp. Notably, transcription of the PEV3 full-length cDNA clone in yeast cells recapitulated the generation of nicks in dsRNA produced *de novo*. However, no results were obtained that could reliably mention the presence of double-stranded RNA as a replicant. These results indicate that the yeast heterologous expression system technology can be a new method to analyze the replication and function of endornaviruses harboring large RNA genomes.

## 2. Materials and methods

### 2.1. Strain and culture conditions

*Phytophthora* rot of asparagus, strain CH98ASP059 (*Phytophthora* sp.), a PEV2 and PEV3 co-staining strain isolated in Toyama Prefecture, Japan, was incubated at 25°C on V8A plate medium. The strains were then cultured in V8 liquid medium at 25°C for 1 week for total RNA and dsRNA extraction.

The yeast strain BY4742 (*MATα, his3Δ, leu2Δ, lys2Δ, ura3Δ*) provided by National BioResource in Japan, was used for the construction of the full-length cDNA clone. YPAD (1% yeast extract, 2% polypeptone, 2% glucose, 0.04% adenine sulfate) was used as nutrient-rich medium, and standard yeast medium (Sherman, 2002) was used as dropout medium. Synthetic complete medium without leucine (SC-Leu) and Synthetic complete medium without uracil (SC-Ura) were used as nutrient dropout media according to standard yeast media (Sherman, 2002). For galactose induction experiments, 2% galactose (SGal) was used instead of 2% glucose (SC).

### 2.2. Extraction of total RNA and dsRNA

Mycelia of strain CH98ASP059 were collected from liquid culture, dried using Miracloth (Merck, Darmstadt, Germany) and sterile filter paper, and stored at −80°C. According to the manufacturer’s recommendation, total RNA was extracted from 0.1 g of mycelia using the RNeasy Plant Mini Kit (QIAGEN, Hilden, Germany). For dsRNA purification, a spin column containing cellulose D (Advantec, Tokyo, Japan) was used (Okada et al., 2015). Mycelia (0.1 g dry weight) were ground in 500 μL of extraction buffer (100 mM NaCl, 10 mM Tris–HCl pH 8.0, 1 mM EDTA, 1% SDS, and 0.1% (v/v) *β*-mercaptoethanol), then mixed with equal volumes of phenol-chloroform-isoamyl alcohol (25:24:1). The aqueous phase containing total nucleic acids was mixed with ethanol (final concentration 16%), and dsRNA was selectively recovered by spin column method. Finally, dsRNA was precipitated by ethanol and stored at −80°C. Quantification of total RNA and dsRNA was confirmed by agarose gel electrophoresis.

Yeast transformants were incubated in 50 mL of SC-Leu or SC-Ura at 30°C for about 16 h (h) with shaking (140 rpm) for RNA extraction. Yeast cells were harvested by centrifugation at 1,000x g for 5 min at 4°C. The collected yeast cells were resuspended in 50 mL of ice-cold sterile water. The cells were then centrifuged in a swinging bucket rotor at 1,000× g at 4°C for 5 min. This step was repeated several times. The resulting yeast cells were crushed with liquid nitrogen, and total RNA was extracted using the RNeasy Plant Mini Kit (QIAGEN, Hilden, Germany) according to the manufacturer’s recommendation. The obtained total RNA was concentrated by ethanol precipitation and stored at −80°C.

### 2.3. Identification of the 3′ end sequence of 1.0 kb-nicks

Rapid amplification of cDNA ends (RACE) was performed to identify the 3′ end of PEV2 and PEV3 nicks. Total RNA extracted and purified from strain CH98ASP059 was polyadenylated by poly (A) tailing reaction before use. The polyadenylated total RNA was used as a template and reacted with the SMARTer® RACE 5′/3’ Kit (Takara Bio USA, Inc., Mountain View, CA, US) according to the manufacturer’s protocol. After amplifying the 3′ end region of the PEV2 and PEV3 nick sequences, they were TA cloned into the pGEM-T easy vector (Promega, Wisconsin, USA) and sequenced by the Sanger method. The primer pairs used are listed in Supplementary Table S1.

### 2.4. Northern blotting analysis of PEV2 and PEV3

**Figure 1:**
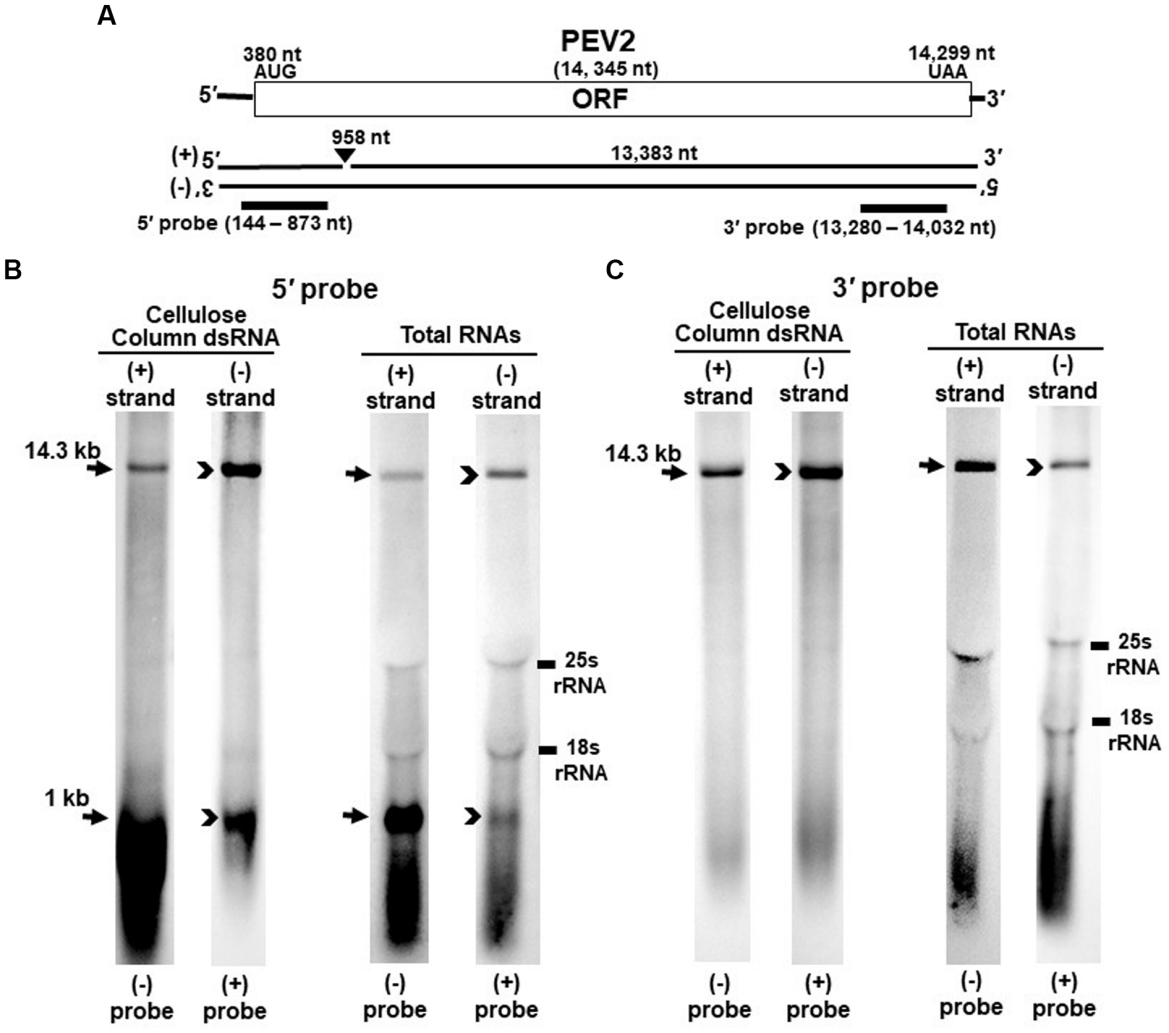
Detection of PEV2 full-length genome and its derived nick fragments in purified dsRNA or total RNA of strain CH98ASP059 by northern blot hybridization. 250 ng of dsRNA and 20 μg of total RNA were heat-denatured and applied. Positive or negative strands were detected separately using DIG-labeled strand specific riboprobes. **(A)** Whole genome map of PEV2 and the location of the riboprobes. **(B)** Detection by the PEV2-5′ probe. With the PEV2-5′ probe, the 14.3 kb full-length genomic RNA and 1 kb nick fragment were detected in both positive and negative strands. **(C)** Detection by the PEV2-3′ probe. Only the 14.3 kb full-length genomic RNA was detected by the PEV2-3′ probe. In the case of total RNA, 25S rRNA and 18S rRNA were detected nonspecifically.

**Figure 2:**
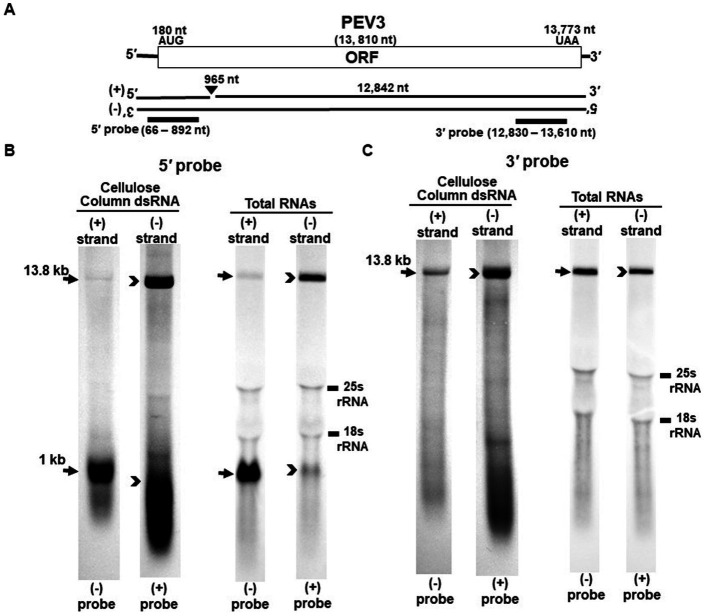
Detection of the PEV3 full-length genome and its derived nick fragments in purified dsRNA or total RNA of strain CH98ASP059 by northern blot hybridization. The detection was performed under the same conditions as for PEV2. **(A)** Whole genome of PEV3 and the position of the riboprobes. **(B)** Detection by PEV3-5′ probe. Using the PEV3-5′ probe, the 13.8 kb genomic RNA and 1 kb nick structure were detected in both positive and negative strands. **(C)** Detection by the PEV3-3′ probe. The PEV3-3′ probe detected the complementation of only the 13.8 kb full-length genomic RNA. In the case of total RNA, 25S rRNA and 18S rRNA were detected nonspecifically.

For northern blotting analysis, 250 ng of dsRNA and 20 μg of total RNA extracted from strain CH98ASP059 or yeast transformants were heat-denatured, separated on 0.8% agarose 3-(N-morpholino) propanesulfonic acid (MOPS) gel containing 6% formaldehyde, and transferred by capillary blotting onto a positively-charged nylon membrane (Roche, Basel, Switzerland) (Moriyama et al., 1999). After UV cross-linking, prehybridization and hybridization were performed in hybrisolution (5× SSPE, 5× Denhardt’s, 0.5% SDS, 0.1 mg/mL denatured salmon testis DNA). A total of 8 probes were used to detect PEV2 and PEV3 genomes: (+) and (−) PEV2-5′ (nt 144–823) and PEV2-3′ probes (nt 13,280–14,032) (Figure 1A), as well as (+) and (−) PEV3-5′ (nt 66–892) and PEV3-3′ probes (nt 12,830–13,610) (Figure 2A) that were prepared to specifically detect the positive and negative strands, respectively, in the genomic regions of each endornavirus. The probe sequences used for hybridization (PEV2-5′ and PEV2-3′ probes (Figure 1A), and PEV3-5′ and PEV3-3′ probes) were amplified by reverse transcription PCR (RT-PCR) using total RNA extracted from CH98ASP059 as a template, and with Superscript IV reverse transcriptase (Invitrogen, Massachusetts, USA) according to the manufacturer’s instructions. PCR using Go-Taq (Promega, Wisconsin, USA) was then performed under the following conditions with primers that are listed in Supplementary Table S1: denaturation for 1 min at 94°C, and 35 cycles of PCR (98°C for 10 s, 55°C for 30 s, and 72°C for 1 min) followed by a final extension step at 72°C for 5 min. The amplified PCR products were gel purified and cloned into the pGEMT-easy vector (Promega).

According to the manufacturer’s protocol, the riboprobes were prepared using the DIG RNA Labeling Kit (SP6/T7) (Roche, Basel, Switzerland). After the plasmids were linearized with the appropriate restriction enzymes (T7 promoter: NcoI, SP6: SalI), each riboprobe was obtained by run-off transcription. Then, DNA probes were prepared by amplification of DIG-labeled PCR products by PCR using the plasmids mentioned above as templates according to the protocol provided with the PCR DIG Labeling Mix (Roche, Basel, Switzerland). The hybridization was performed in a solution containing specific DIG-labeled RNA or DNA probes for PEV2 or PEV3 sequences for 16 h at 65°C.

Detection of bands using the DIG system was performed using a modification of the manufacturer’s recommended protocol. After hybridization, the membrane was washed twice at 65°C for 5 min in low stringency buffer (2× SSC, 0.1% SDS) and twice for 15 min in high stringency buffer (0.1% SSC, 0.1% SDS). For detection, the membrane was washed in maleic acid wash buffer (1× maleic acid, 0.3% Tween20, pH 7.0) for 2 min, followed by blocking in DIG blocking buffer (Roche, Basel, Switzerland) for 1.5 h. Anti-Digoxigenin-AP, Fab fragments from sheep (Roche, Basel, Switzerland) diluted 5,000-fold in DIG blocking buffer was added and shaken for another 30 min. The membrane was washed again with maleic acid wash buffer for 15 min and repeated twice. The membrane was then incubated in DIG detection buffer (Tris–HCl, pH 9.5, 0.1 M NaCl) for 2 min. Then, ready-to-use CDP-Star^®^ (Roche, Basel, Switzerland) was added directly to the membrane and allowed to stand in the dark for 5 min, and the signal was detected by Ez-Capture MG (ATTO, Tokyo, Japan).

### 2.5. S1 nuclease treatment

DsRNA and total RNA were dissolved in 2.0× or 0.2× SSC, and ssRNA was selectively digested by S1 nuclease (10 U) for 15 min at room temperature.

### 2.6. Full-length cDNA cloning of PEV2 and PEV3 in yeast cells

First, five and six fragments were amplified by RT-PCR using the PEV2 and PEV3 RNA genomes as templates, respectively, with the high-fidelity PrimeSTAR^®^ Max DNA Polymerase (TaKaRa, Shiga, Japan). The following conditions and primers that are listed in Supplementary Table S1 were used: denaturation for 1 min at 94°C, and 35 cycles of PCR (98°C for 10 s, 55°C for 10 s, and 68°C for 2 min) followed by a final extension step at 68°C for 5 min. The PCR products were then purified by the gel extraction method. Then, the fragments were assembled and cloned in the correct order into a shuttle vector by DNA homologous recombination events that occur between overlapping sequence regions of each fragment, by taking advantage of the high DNA recombination activity of yeast cells using lithium acetate method (Gietz et al., 1995). The yeast strain BY4742 (*MATα, his3Δ, leu2Δ, lys2Δ, ura3Δ*) was cultured in YPAD liquid medium, and the recovered yeast cells were transformed with 25 ng EcoRI- and SmaI-linearized shuttle vectors (pRST425 or pRST426) and 250 ng each of five PEV2 fragments (A ~ E) or six PEV3 fragments (A ~ F) that were previously amplified by RT-PCR.

The transformed yeast were streaked on drop-out medium plates (SC-Leu or SC-Ura), then single colonies were picked for total nucleic acid extraction, and PCR was performed using primers that specifically amplify PEV2 or PEV3.

### 2.7. Growth tests of PEV2- or PEV3-transformed yeast cells

We compared the growth of BY4742 cells transformed with vectors harboring the galactose-inducible promoter fused with either PEV2 or PEV3 genome (pYES2-PEV2 and pYES2-PEV3, respectively) with that of control (pYES2-Empty). For the dilution drop tests, yeast cells were cultured first on SC-Ura agar medium at 30°C for 2 days, then inoculated into 2 mL of SC-Ura liquid medium and precultured at 30°C with shaking overnight at 140 rpm. The precultured yeast cells were inoculated into 50 mL of SGal-Ura liquid medium at OD_600_ = 0.1 and then incubated at 30°C with shaking at 140 rpm until OD_600_ = 1.0. The cells were subsequently serially diluted ten times in distilled water (DW), and diluted cells were dropped at a volume of 10 μL each onto SGal-Ura (containing 2% galactose) agar medium, which induces heterologous expression, and SC-Ura (containing 2% glucose) agar medium as control. Then their growth was examined after incubation at 30°C for 2 days. One mL of the culture suspension was collected every 4 h from 0 to 12 h after inoculation, and every 12 h thereafter. The number of viable cells, turbidity (OD_600_), and cell size (after 24 h of incubation) were measured by Countstar (ABER, Aberystwyth, UK).

### 2.8. Enhanced green fluorescence protein (EGFP) fusion protein constructs and fluorescence microscopy

Plasmids expressing PEV2- or PEV3-EGFP fusion protein in *S. cerevisiae* strain BY4742 were constructed based on pRST426, which had a strong *TDH3* promoter and terminator cassette as per our previous report (Urayama et al., 2016). The BY4742 strain was transformed with the shuttle vectors pRST426-EGFP, pRST426-PEV2-NEGFP, pRST426-PEV2-CEGFP, pRST426-PEV3-NEGFP or pRST426-PEV3-CEGFP. Transformants were streaked to obtain single colonies and incubated on SC-Ura agar plates at 30°C for 3 days. A portion of the single colonies was suspended in DW, and the cells and GFP fluorescence were observed at 1,000x magnification using an optical microscope (Olympus IX71, Tokyo, Japan) and differential interference contrast (DIC) optics.

### 2.9. Statistics analysis

The data of yeast cell diameter were subjected to analysis of variance. Statistical significance is considered when *p* < 0.05. Cell diameter measurements after 24 h of incubation were averaged and performed three times. Differences between means of different treatments were determined using the Tukey–Kramer test at *p* < 0.01.

## 3. Results

### 3.1. Detection of nick structures in PEV2 and PEV3

Nick structures in the 5′ region of the positive strand of dsRNA replicative intermediate in plant endornaviruses were found by northern blotting (Fukuhara et al., 1995), while those in Phytophthora endornavirus 1 (PEV1) were suggested by RNA ligase-mediated RACE sequencing of the 3′-terminal of the RNA (Hacker et al., 2005). In this paper, we performed northern blotting of total RNA and dsRNA from PEV2 (Figure 1) and PEV3 (Figure 2) infecting *Phytophthora* rot of asparagus (strain CH98ASP059) to determine whether nick structures were also present.

We used eight probes to detect high molecular weight bands from purified dsRNA and total RNA that presumably corresponded to the whole genome: a 14.3 kb band from PEV2 (Figures 1B,C) and a 13.8 kb band from PEV3 (Figures 2B,C). Moreover, 1 kb bands were also detected in both endornaviruses, which indicated the presence of nick structures (Figures 1B, 2B). However, these 1 kb bands were detected only when 5′ probes were used for both PEV2 (Figure 1B, black arrows) and PEV3 (Figure 2B, black arrows). As in previous reports of plant endornavirus nick structures, a strong signal in the positive strand of the nick structure was detected by the (−) probe, which detects the positive strand near the 5′ end (Fukuhara et al., 1995). However, our results also showed a signal in the negative strand of the nick structure as detected by the (+) probe. When only 3′ probes for both strands were used, no signal was detected in either strand of PEV2 (Figure 1C) and PEV3 (Figure 2C). These results were similar to those previously reported for plant endornaviruses such as OsEV and BPEV (Fukuhara et al., 1995; Okada et al., 2011), indicating site-specific nick structures near the 5′ terminal in PEV2 and PEV3. These nick structures were also detected by DNA probes (Supplementary Figure S1).

Notably, in PEV2, a 1 kb signal was also detected in the negative strand using the PEV2-5′ (+) probe that specifically detects the negative strand near the 5′ terminal (Figure 1B, black arrowheads), which has not been shown thus far in other plant endornaviruses. The intensity of the 1 kb signal of the positive strand was stronger than that of the negative strand in PEV2. In PEV3, a 1 kb band in the negative strand was also observed, albeit at a weaker signal intensity (Figure 2B, black arrowheads).

The specific position of the 3′-nick terminal was determined by the 3′-terminal RACE method. The 3′-terminal of the nicks were found to be located at nt 958 in PEV2 by sequencing four independent clones and at nt 965 in PEV3 by sequencing five independent clones. These results corresponded to the northern blot analyses and showed that the nick structures were detected around 1,000 nt from the 5′-terminal in PEV2 and PEV3, suggesting that PEV2 and PEV3 have nick structures at similar positions as in previously reported endornaviruses. The 5′-terminal RACE method was also attempted as previously shown for OsEV and BPEV, which failed due to the relatively long 3′ fragment of PEV2 and PEV3. It is possible that the different results were obtained because purified dsRNA was used as a template for cloning nicks in OsEV and BPEV, while total RNA was used as a template for PEV2 and PEV3 in this study. However, since full-length genomic RNA was detected by the northern blot analysis using probes that detected the 5′-terminal sequence of PEV2 or PEV3 (Figures 1B, 2B), the 5′-terminal sequence of each RNA genome was assumed to be amplified by the 5′-terminal RACE method and our conjecture on its failure was considered to be reasonable.

### 3.2. Molecular characterization of the 1 kb nick fragments

**Figure 3:**
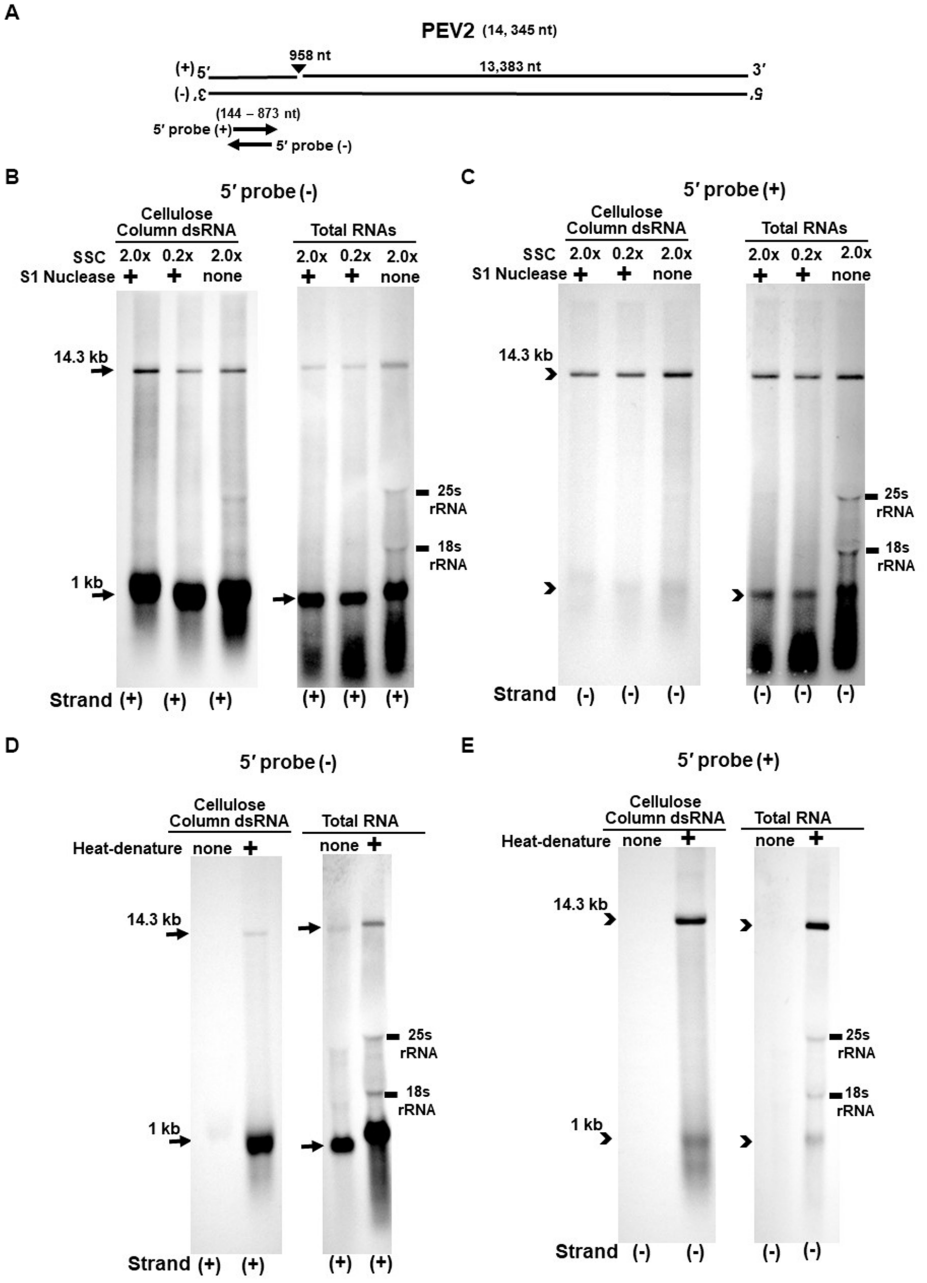
**(A)** Whole genome diagram of PEV2 and location of riboprobes. **(B,C)** Detection of PEV2 genomic RNA and nick fragments by northern blot hybridization after S1 nuclease treatment. **(B)** Specific detection of positive strand by PEV2-5′ (−) probe. Left lane: 2.0x SSC, with S1 nuclease treatment; middle lane: 0.2x SSC, with S1 nuclease treatment; right lane: 2.0x SSC, without S1 nuclease treatment. **(C)** Specific detection of negative strand by PEV2-5′ (+) probe. Left lane: 2.0x SSC, with S1 nuclease treatment; middle lane: 0.2x SSC, with S1 nuclease treatment; right lane: 2.0x SSC, without S1 nuclease treatment. **(D,E)** Comparison of detection patterns of PEV2 genomic RNA and nick with and without heat denaturation. Heat denaturation of dsRNA or total RNA was performed at 65°C for 10 min, followed by 2 min on ice. The untreated samples were directly applied after dissolving in RNA loading buffer. **(D)** Specific detection of positive strand by PEV2-5′ (−) probe. Left lane: without heat denaturation; right lane: with heat denaturation. **(E)** Specific detection of the negative strand by the PEV2-5′ (+) probe. Left lane: without heat denaturation; right lane: with heat denaturation.

**Figure 4:**
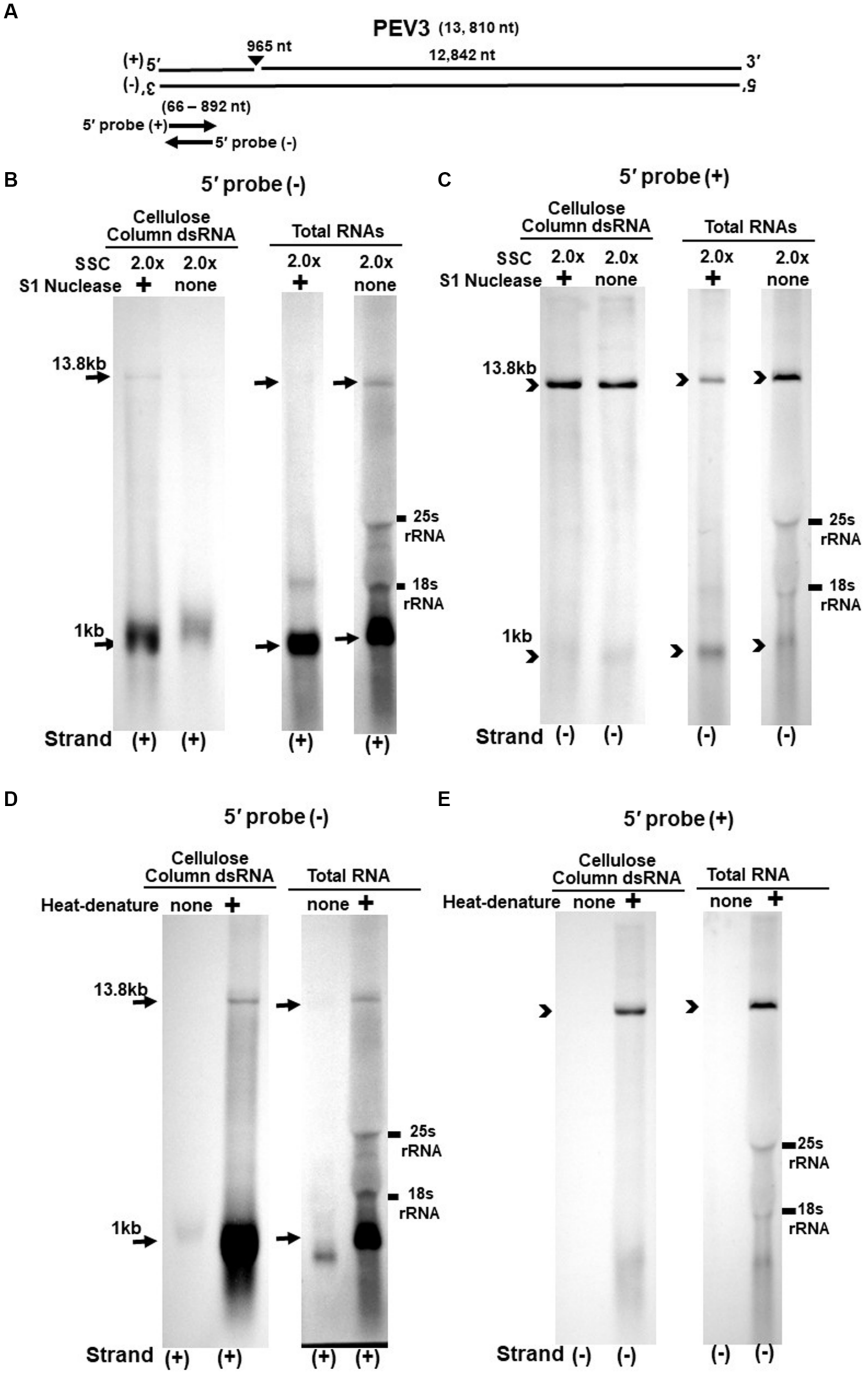
**(A)** Whole genome diagram of PEV3 and location of riboprobes. **(B,C)** Detection of PEV3 genomic RNA and nick fragments by northern blot hybridization after S1 nuclease treatment. **(B)** Specific detection of positive strand by PEV3-5′ (−) probe. Left lane: 2.0 x SSC, with S1 nuclease treatment; middle lane: 0.2 x SSC, with S1 nuclease treatment; right lane: 2.0 x SSC, without S1 nuclease treatment. **(C)** Specific detection of negative strand by PEV3-5′ (+) probe. Left lane: 2.0x SSC, with S1 nuclease treatment; middle lane: 0.2x SSC, with S1 nuclease treatment; right lane: 2.0x SSC, without S1 nuclease treatment. **(D,E)** Comparison of detection patterns of PEV3 genomic RNA and nick with and without heat denaturation. Heat denaturation of dsRNA or total RNA was performed at 65°C for 10 min, followed by 2 min on ice. The untreated samples were directly applied after dissolving in RNA loading buffer. **(D)** Specific detection of positive strand by PEV3-5′ (−) probe. Left lane: without heat denaturation; right lane: with heat denaturation. **(E)** Specific detection of the negative strand by the PEV3-5′ (+) probe. Left lane: without heat denaturation; right lane: with heat denaturation.

To investigate in detail the genomic structure of the nicks in PEV2 and PEV3 we used the probes for detecting the nicks (PEV2-5′ (+, −) and PEV3-5′ (+, −) probes) following S1 nuclease treatment (Figures 3A, 4A).

The S1 nuclease tests were performed under two conditions: a high salt concentration of 2.0x SSC, which strengthens the binding between each strand of dsRNA, and a low salt concentration of 0.2x SSC, which relaxes it (Figures 3B,C, 4B,C).

When the PEV2-5′ (−) probe was used following treatment with S1 nuclease, both bands corresponding to the 14.3 kb PEV2 genome and 1 kb nick fragment were detected in the dsRNA and total RNA (Figure 3B). On the other hand, when the PEV2-5′ (+) probe was used, the 14.3 kb band was exclusively detected in the purified dsRNA samples, and the 1 kb nick fragment was detected only rarely (Figure 3C, left side). However, a 1 kb nick fragment was detected in the total RNA sample (Figure 3C, right side), indicating that negative-stranded 1 kb nick fragments occurred in small amounts. In any case, the presence of 1 kb nick fragments after S1 nuclease treatment and denaturing gel electrophoresis suggested that dsRNA forms of PEV2 have a nick structure in the positive strand (Figure 3A). Similar results were obtained in PEV3; the PEV3-5′ (−) probe detected a band of 13.8 kb that corresponded to the positive strand of the PEV3 genome and a 1 kb nick fragment in the positive strand, although the signal was relatively weak (Figure 4B). The PEV3-5′ (+) probe detected exclusively the 14.3 kb band corresponding to the negative strand of the genome and only a tiny amount of the 1 kb nick fragment of the negative strand (Figure 4C).

Since the previous experiments showed that the nick structure in the positive strand was more strongly detected (higher quantity) than that in genomic RNA by northern blotting (Figures 1, 2), the nick structure in the positive strand may also exist as ssRNA independent of genomic RNA. To clarify this hypothesis, non-denatured RNA was electrophoresed and under conditions allowing ssRNA to be preferentially detected, we investigated the nick structure using 5′-terminal strand-specific riboprobes for PEV2 (Figures 3D,E) and PEV3 (Figures 4D,E). In total RNA from PEV2, the 14.3 kb genomic band and the 1 kb nick fragment band were detected by the PEV2-5′ (−) probe (Figure 3D right side). On the other hand, no band was detected by the PEV2-5′ (+) probe, which specifically detects the negative strand, under non-denaturing condition (Figure 3E, right side). In the case of PEV3, the 13.8 kb positive strand genomic band and the 1 kb nick fragment band were detected only with the PEV3-5′ (−) probe (Figure 4D, right side). This result suggests that the genomic and nick RNA components of PEV2 and PEV3 exist as ssRNA(+) in the host cells, while the negative strand genomic RNA and nick fragments of both viruses exist only as dsRNA. This series of experiments indicated that the positive-stranded 1 kb nick fragment exists independently of dsRNA in host cells, while the negative-stranded 1 kb nick fragment exists only in dsRNA form.

### 3.3. Full-length cDNA clones of PEV2 and PEV3 in yeast cells

**Figure 5:**
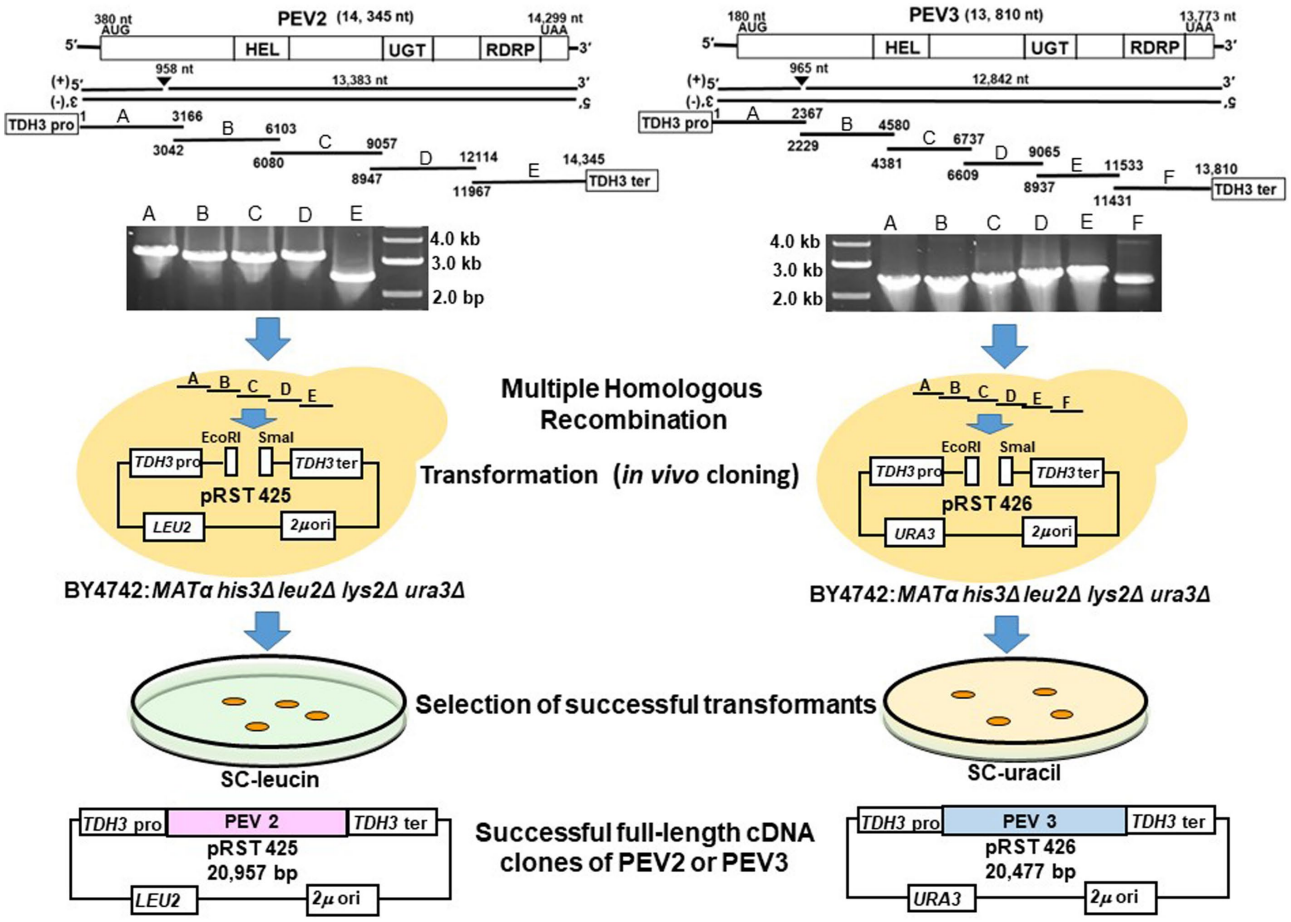
Schematic diagram of the construction of full-length cDNA clones of PEV2 and PEV3 in yeast cells. Five overlapping fragments (A–E) of PEV2 and six overlapping fragments (A–F) of PEV3 were amplified by RT-PCR using total RNA extracted from CH98ASP059 as template, and electrophoresis of the amplified products are shown. These fragments and the shuttle vectors, pRST425 and pRST426, linearized by double digestion with EcoRI and SmaI, respectively, were simultaneously transformed into strain BY4742 and linked by homologous recombination (HR) in yeast cells. Transformants were selected by nutrient requirement markers (*LEU2*: leucine selection marker for PEV2 and *URA3*: uracil selection marker for PEV3) linked to the shuttle vectors. Finally, the constructs of these two full-length cDNA clone shuttle vectors are shown.

Twelve colonies that were selected for analysis from the drop-out SC-Leu or SC-Ura medium plates harbored the full-length PEV2 and PEV3 cDNAs cloned in pRST425 and pRST426, respectively (Figure 5, Supplementary Figure S2). Less frequently, colonies that did not contain the desired sequence were obtained, which were transformants of empty shuttle vectors possibly due to incomplete restriction enzyme treatment. These results showed that highly efficient constructions of full-length cDNA clones of PEV2 and PEV3 were achieved using DNA homologous recombination in yeast cells.

### 3.4. Transcript analysis of PEV2 and PEV3 genomic RNA expressed in yeast cells

**Figure 6:**
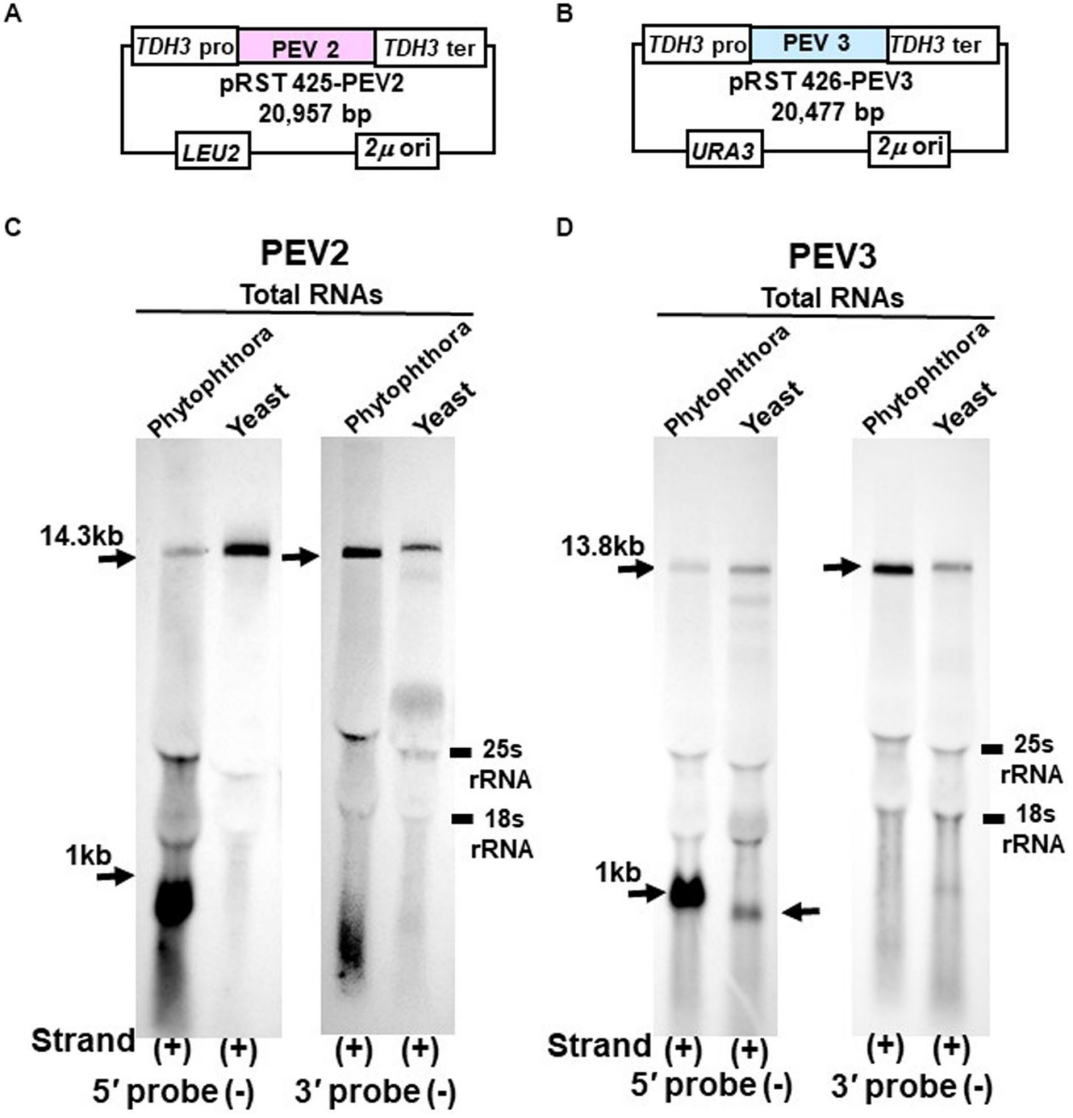
Northern blot hybridization of total RNA extracted from PEV2 or PEV3 from *Phytophthora* sp., strain CH98ASP059, and total RNA extracted from yeast carrying full-length PEV2 cDNA or full-length PEV3 cDNA clones. **(A)** Construct of pRST425-PEV2. **(B)** Construct of pRST426-PEV3. **(C)** Detection of positive strands of PEV2-derived RNA by PEV2-5′ or PEV2-3′ probe. Left lane: *Phytophthora* sp., strain CH98ASP059; right lane: yeast strain with pRST425-PEV2 (full-length PEV2 cDNA clone). **(D)** Detection of positive strands of PEV3-derived RNA by PEV3-5′ or PEV3-3′ probe. Left lane: *Phytophthora* sp., strain CH98ASP059; right lane: yeast strain carrying pRST426-PEV3 (full-length PEV3 cDNA clone).

Using yeast cells transformed with pRST425-PEV2 (Figure 6A) and pRST426-PEV3 (Figure 6B), northern blotting was performed to analyze transcripts of PEV2 (Figure 6C) and PEV3 (Figure 6D) genomic RNA transcribed by RNA polymerase II under the control of the constitutive *TDH3* promoter.

In the yeast transformants of pRST425-PEV2, a band of 14.3 kb, which corresponded to the full-length PEV2 genome, was detected with both PEV2-5′ (−) and PEV2-3′ (−) probes (Figure 6C). On the other hand, in the yeast transformants of pRST426-PEV3, a band around 1 kb in size was detected in addition to the full-length band of 13.8 kb when the PEV3-5′ (−) probe was used (Figure 6D, left side). When the PEV3-3′ (−) probe was used, only a band of 13.8 kb in size was detected. The results of a parallel experiment using total RNA from *Phytophthora* were similar to those shown in Figures 1B,C, 2B,C. To determine the size of nick-like RNA occurring in yeast cells, we performed 3’RACE to PEV3 heterologously expressed in yeast (Supplementary Figure S3). After polyadenylated by poly (A) tailing reaction, an 856 nt amplified band whose size was 109 nt shorter than the PEV3 nick occurred in native host, strain CH98ASP059 (nt 965) was observed in heterologously PEV3 expressed yeast cells (YP3) (Supplementary Figure S3). Without poly (A) tailing reaction, no bands were amplified in the strain CH98ASP059 ASP nor YP3 when PEV3-66 primer located in a 5′ -nick fragment was used (Supplementary Figure S3). Using primer PEV3-12830 which is located near the 3′ terminus, a band of 980 bp was amplified with polyadenylated transcripts of ASP and YP3, whose 3′ terminuses were polyadenylated. This result showed that the RNA of nt 856 generated by YP3 did not have Poly (A) and might not be generated by termination of transcription by RNA Polymerase II.

These results indicate that the *TDH3* promoter driven in yeast cells can complete transcription of the PEV2 and PEV3 full-length RNA genomes. Besides, a 1 kb-sized nick product was detected in pRST426-PEV3-transformed cells (Figure 6D, left side), albeit less intense and smaller than that from the natural host *Phytophthora* spp.

### 3.5. Overexpression of PEV2 and PEV3 inhibits yeast growth

**Figure 7:**
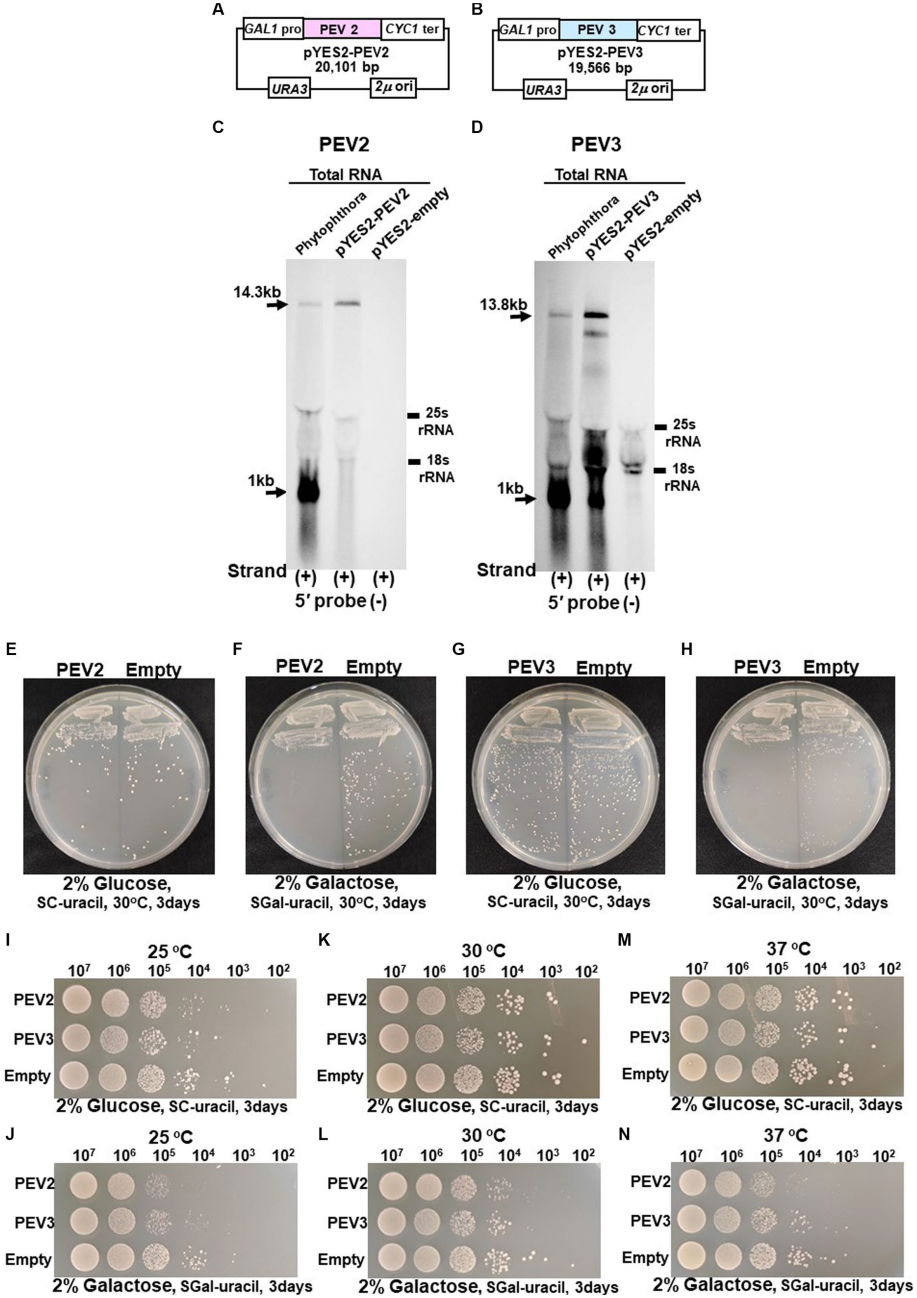
**(A)** Construct of pYES2-PEV2. **(B)** Construct of pYES2-PEV3. **(C)** Detection of positive strands of PEV2-derived RNA by PEV2-5′ or PEV2-3′ probe. Left lane: *Phytophthora* sp., strain CH98ASP059; middle lane: yeast strain carrying pYES2-PEV2 (full-length PEV2 cDNA clone); right lane: yeast strain carrying pYES2-Empty (empty vector) (Control). **(D)** Detection of positive strands of RNA derived from PEV3 sequence by PEV3-5′ probe. Left lane: *Phytophthora* sp., strain CH98ASP059; middle lane: yeast strain carrying pYES2-PEV3 (full-length PEV3cDNA clone); right lane: yeast strain carrying empty vector (pYES2-Empty) (Control). **(E,F)** Comparison of growth of single colonies of pYES2-PEV2 and pYES2-Empty yeast transformants. **(E)** Uracil drop-out, non-induced medium containing 2% glucose. **(F)** Uracil drop-out, induced medium containing 2% galactose. **(G,H)** Comparison of growth of single colonies of pYES2-PEV3 and pYES2-Empty yeast transformants. **(G)** Uracil drop-out, non-induced medium containing 2% glucose. **(H)** Uracil drop-out, induced medium containing 2% galactose. **(I–N)** Drop tests of pYES2-PEV2, pYES2-PEV3 or pYES2-Empty transformed yeast cells grown at 25°C, 30°C or 37°C. OD_600_: 1.0 = 10^7^ cells/mL was adjusted, then successively diluted 10-fold to 10^2^ cells/mL, then 10 μL of each was dropped. **(I,K,M)** Uracil drop-out, non-induced medium containing 2% glucose. **(J,L,N)** Uracil drop-out, induced medium containing 2% galactose. The strain with empty vector of pYES2 (Empty) was used as control strain. Each test was repeated in triplicate.

We previously reported that high titer of PEV2 and PEV3 in *Phytophthora* fungus severely inhibited hyphal growth (Uchida et al., 2021). To investigate the effect of expression of the full-length PEV2 and PEV3 RNA on yeast cell growth, we generated constructs (pYES2-PEV2 and pYES2-PEV3, respectively) in which the full-length cDNA clones of PEV2 or PEV3 were cloned downstream of the galactose-inducible promoter (*GAL1*) (Figures 7A,B). These constructs were created using multiple homologous recombination as in the cases of pRST425-PEV2 and pRST426-PEV3 (Supplementary Figure S2). A 14.3 kb band from pYES2-PEV2-transformed cells and a 13.8 kb band from pYES2-PEV3-transformed yeast (Figures 7C,D) were detected by northern blot hybridization using the PEV2-5′ (−) and PEV3-5′ (−) probes to assess for PEV2 and PEV3 RNA transcripts transcribed under the control of *GAL1*. A 1 kb nick fragment was also detected as a distinct band in yeast cells harboring pYES2-PEV3 (Figure 7D).

Colony formation on SC-Ura agar medium as well as viable cell counts and turbidity (OD_600_) over time in SC-Ura liquid medium were compared among the three transformants of BY4742 strain harboring pYES2-PEV2, pYES2-PEV3, and pYES2-Empty (for control). On 2% glucose agar medium, no difference in single colony growth was observed between the PEV2- or the PEV3-expressing transformants and the control transformants (Figures 7E,G). On the other hand, in 2% galactose agar medium where the *GAL1* promoter induced downstream gene expression, the PEV2 and the PEV3-expressing transformants had fewer colonies, showed smaller colony size, and had reduced growth as compared to the control transformants (Figures 7F,H).

In addition, we investigated the effects of heterologous expression of PEV2 and PEV3 at low (25°C) and high temperatures (37°C) on yeast cell growth. On SC-Ura agar medium containing 2% glucose, no discernible difference in growth was observed between the three transformants at each temperature (Figures 7I,K,M), but on SC-Ura containing 2% galactose, the growths of the PEV2 and PEV3 transformants were apparently slower than that of the control (Empty) transformants under both temperatures (Figures 7J,L,N).

**Figure 8:**
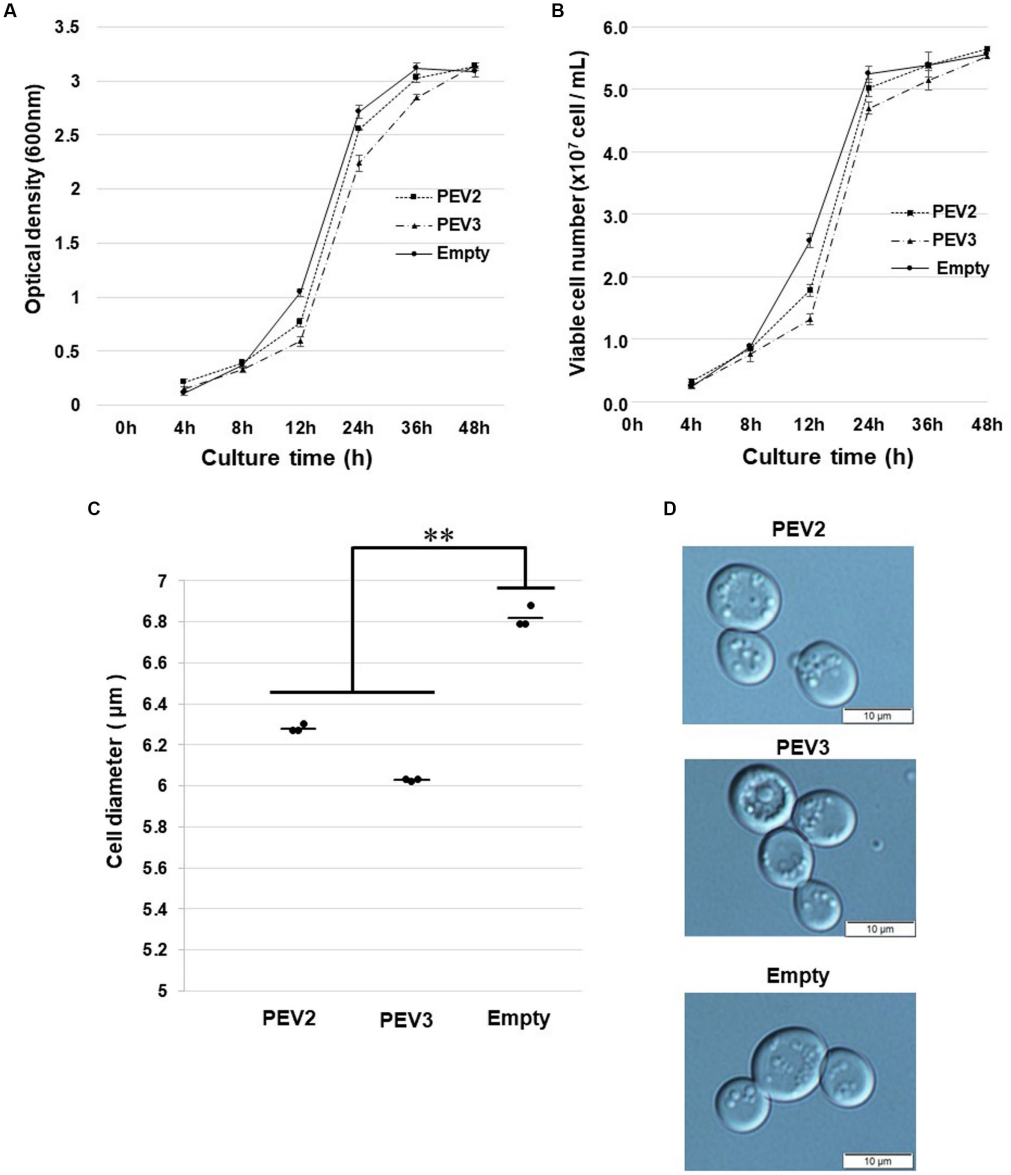
Growth tests of yeast cells with heterologous expression of PEV2 and PEV3 over time following induction with galactose. Yeast cells transformed with pYES2- PEV2 (*GAL1* promoter), pYES2- PEV3 (*GAL1* promoter) or pYES2-Empty were cultured in uracil drop-out, induced liquid medium containing 2% galactose at 30°C. **(A)** Number of viable cells. **(B)** Turbidity (OD_600_) values. Measurements were taken every 4 h from 0 to 12 h and every 12 h from 12 to 48 h. PEV2 (■, dotted line), PEV3 (▲, dashed line) and Empty (●, line). Each value is presented as mean ± standard error (*n* = 3). **(C)** Comparison of cell diameter at 24 h. The dots indicate the mean value for each sample, and the bars indicate the mean value for the test area (*n* = 3). **, *p* < 0.01. **(D)** Yeast cells transformed with PEV2 (top), PEV3 (middle), and Empty (bottom) at 24 h. Photo bar = 10 um. Statistical significance is considered when *p* < 0.05.

In growth tests based on OD_600_ values in SC-Ura liquid medium, the turbidity of PEV2 and PEV3 transformants was significantly lower as compared to control transformants (empty) after 12 h of incubation, but subsequently reached almost identical levels to that of control (Figure 8A). Similar results were obtained by counting the number of viable cells in these three transformants (Figure 8B). After 24 h of incubation at 30°C, the sizes of living cells of PEV2 and PEV3 (average diameter = 6.25 μm and 6.05 μm, respectively; Turkey test: *p* < 0.01) transformants were significantly smaller as compared to the control strain (6.8 μm) (Figure 8C). The observed cell sizes of the empty appeared to be larger than those of PEV2 and PEV3. PEV2, PEV3, and empty cells were also observed under an optical microscope after 24 h of incubation at 30°C. PEV2 and PEV3 cells were apparently uniform in size rather than the empty cells which tended to show varied cell sizes (Figure 8D). These results suggested that the heterologous expression of PEV2 and PEV3 did not induce cell death toxicity in yeast cells, but had inhibitory effects on the growth rates of the cells.

### 3.6. Expression of PEV2- and PEV3-EGFP fusion proteins in yeast

**Figure 9:**
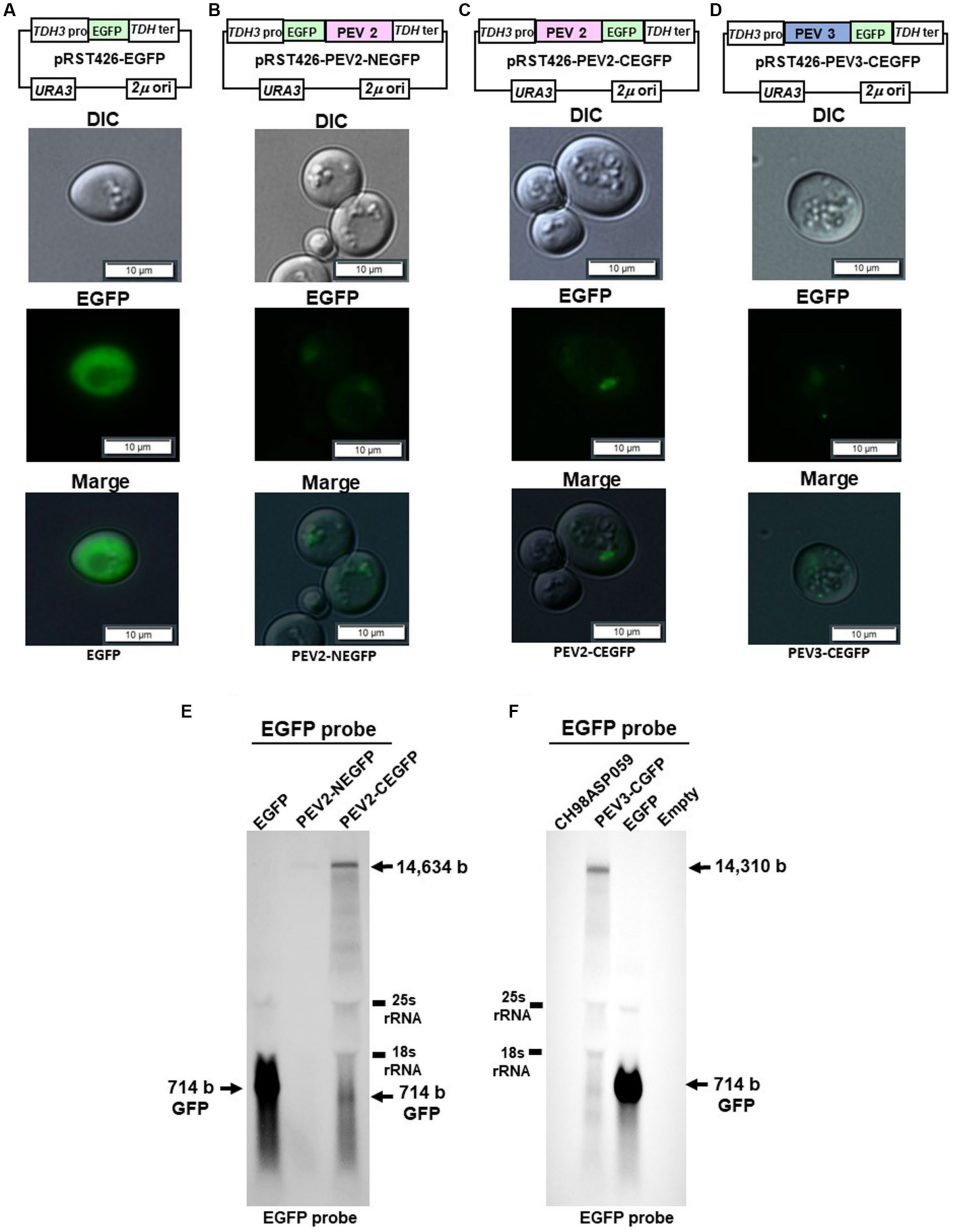
Localization of PEV2- and PEV3-EGFP fusion proteins in yeast cells. The transformed yeast cells were streaked on SC-Ura agar (glucose) and cultured at 30°C for 3 days to form single colonies, which were then suspended in DW and observed under a fluorescence microscope. **(A)** pRST426-EGFP, **(B)** pRST426-PEV2-NEGFP, **(C)** pRST426-PEV2-CEGFP, **(D)** pRST426-PEV3-CEGFP. Image bar = 10 mm. Fusion protein expression constructs were prepared by adding EGFP at the N-terminus of PEV2 ORF [pRST426-PEV2-NEGFP, **(B)**] or C-terminus of PEV2 ORF [pRST426-PEV2-CEGFP, **(C)**], and EGFP at the C-terminus of PEV3 ORF [pRST426-PEV3-CEGFP, **(D)**] downstream of TDH3 promoter. **(E,F)** Northern blot analysis in yeast expressing EGFP fusion proteins. **(E)** Total RNA purified from transformed yeast cells with pRST426-EGFP, pRST426-PEV2-NEGFP, or pRST426-PEV2-CEGFP, **(F)** Total RNA purified from CH98ASP059 (*Phytophthora* sp.), and yeast cells transformed with pRST426-PEV3-CEGFP, pRST426-EGFP, or pRST426-empty were detected by EGFP probe targeting EGFP sequence after blotting. All results showed only the positive strands.

To visually observe the expression of the endornavirus ORF proteins, EGFP fusion expression vectors were constructed by fusing EGFP with the N-terminus of the PEV2 or PEV3 ORF (PEV2-NEGFP and PEV3-NEGFP, respectively), or their C-terminus (PEV2-CEGFP and PEV3-CEGFP, respectively) (Figures 9A–D). These vectors were constructed by multiple homologous recombination of the full-length ORF of PEV2 or PEV3 in yeast cells using pRST426-EGFP, in which EGFP was incorporated under the *TDH3* promoter. In this study, we failed to obtain PEV3-NEGFP constructs despite several attempts (data not shown), which may be due to the toxicity of the fused protein caused by structural perturbation.

Yeast cells carrying the above constructs, which expressed the PEV2- or PEV3-EGFP fusion protein, were cultured for 20 h, and EGFP fluorescence was observed under a fluorescence microscope. Yeast cells that were transformed with the control pRST426-EGFP vector and expressed free EGFP exhibited strong green fluorescence throughout the cytoplasm (Figure 9A). On the other hand, very weak green fluorescence was observed in yeast cells expressing PEV2-NEGFP, possibly due to the very low expression level of the PEV2-NEGFP fusion protein (Figure 9B). In contrast, PEV2-CEGFP showed some bodies in cells (Figure 9C). PEV3-CEGFP also exhibited a conspicuous localized dot-like green fluorescence (Figure 9D).

To examine the level of transcripts produced from the EGFP fusion constructs, northern blotting using the EGFP sequence as a probe was performed (Figures 9E,F). In cells transformed with control pRST426-EGFP, a strong band of 714 bp was detected, whereas weak bands of around 14.5 kb and 714 bp were detected in cells transformed with pRST426-PEV2-NEGFP and pRST426-PEV2-CEGFP (Figure 9E). Further, the intensity of a 1.5 kb band was much weaker in PEV2-NEGFP-expressing cells as compared to that in cells with PEV2-CEGFP (Figure 9E), in accordance with the observed difference in fluorescence intensity (Figures 9B,C). Northern blot analysis for PEV3-CEGFP showed that the intensity of the signal was comparable to that of PEV2-CEGFP (Figure 9F), consistent with the results of fluorescent microscopy (Figure 9D).

## 4. Discussion

The presence of nick structures has been reported in many plant endornaviruses and has been analyzed by northern blot analysis, which allows visualization of the molecular morphology of the RNA genomes by detecting fragment bands of specific sizes (between 0.9 and 2.7 kb), or by sequencing using RACE (Fukuhara et al., 1995; Pfeiffer, 1998; Moriyama et al., 1999; Okada et al., 2011). In this report, northern blot analysis of endornaviral RNA samples after heat denaturation detected bands of 14.3 kb and 1 kb from PEV2, and 13.8 kb and 1.0 kb from PEV3 (Figures 1B, 2B). Nick fragments of 1 kb in size were more abundant in the positive strand of both viral genomes than in the negative strand. These results showed that the nick structure is conserved in endornaviruses infecting *Phytophthora* as well as in other plant endornaviruses such as rice and bell pepper in terms of similar genomic positions and structures.

On the other hand, northern blot analysis of non-denatured total RNA detected a 14.3 kb positive-stranded ssRNA and a 1 kb positive-stranded fragment in PEV2, and a 13.8 kb positive-stranded ssRNA and 1 kb positive-stranded fragment in PEV3 (Figures 3D, 4D), although no negative-stranded RNA was detected in PEV2 and PEV3 (Figures 3E, 4E). These results suggest that PEV2 and PEV3 exist in host cells as dsRNA replication intermediates and as positive-stranded RNA.

In this study, we established a yeast heterologous expression system on a reverse genetics basis to investigate the effects of endornavirus infection on the host and the nicks that endornaviruses produce in host cells. We successfully constructed full-length cDNA clones of endornaviruses using yeast DNA recombination technology, which allows seamless cloning without restriction enzymes (Figure 5). At first, we tried to clone full-length PEV2 and PEV3 cDNA by transformation into *E. coli* without success. One of the difficulties often encountered in full-length cDNA cloning of potyviruses with long ssRNA(+) genomes is that potential prokaryotic promoter activity in the viral sequence can interfere with the cDNA cloning process in *E. coli* (Yang et al., 1998; López-Moya and García, 2000). Similar interference may have occurred with endornaviruses.

In contrast, construction of heterologous expression systems of PEV2 and PEV3 genomic RNA in yeast was successful. Notably, the PEV3-derived RNA transcripts showed the reproduction of nick fragments in yeast cells, and a significant increase in nick production was observed under galactose induction (Figure 7D) as compared to the constitutive expression (Figure 6D). These results suggest that the increase in the amount of PEV3 full-length ssRNA transcripts due to the enhanced transcriptional activity of RNA polymerase II is accompanied by a consequent increase in the production of nick fragments. It was shown that the nick may arise not only as a broken structure of dsRNA, but also through the transcription process of ssRNA, providing us with important clues to elucidate the mechanisms by which nick structures are generated. Well-known examples of self-cleavage of RNA transcripts are shown by the self-cleavage activities of the CTDs (carboxyl-terminal domains) of RNA polymerase I (Kuhn et al., 2007) and RNA polymerase II (Hsin and Manley, 2012). Investigation of PEV2 or PEV3 transcript expressed in a CTD-deficient *rpa12* mutant strain of RNA polymerase I or in a CTD-deficient *rpo21* mutant strain of RNA polymerase II would provide additional information on nick production. Alternatively, construction of full-length clone shuttle vectors with mutated sequences in the C-terminal regions of the predicted RdRPs of PEV2 and PEV3 would allow the examination of the presence of cleavage activities in these motif regions.

In addition, there are two other hypotheses for the generation of nick. One is the possibility that ribozyme-like secondary structures exist near the nicks’ cleavage positions and that intracellular autonomous cleavage occurs. For example, the ribozyme activity of hepatitis delta virus has been used to recapitulate the complete 3′ end of RNA viruses (Thill et al., 1991). It is unclear whether there is a secondary structure near the sequence where the nick occurs in PEV2 and PEV3 that could give rise to ribozyme activity. The other possibility is that the endornaviral genome encodes a putative RNA nicking endonuclease that cleaves at a specific position. A putative RNA nicking endonuclease may exist as one of the post-processing proteins among the more than 4,000 aa ORF encoded by endornaviruses. Potyviruses and picornaviruses encoding a single ORF are known to self-cleave polyproteins by proteases encoded in their genome (Carrington and Dougherty, 1987; Hellen et al., 1989). Future studies should analyze whether the proteases and the nicking endonucleases produced after the processing of polyproteins in endornaviruses also have functions in self-modification of proteins and genomic RNA.

Toward this goal, we fused EGFP to the polyproteins encoded by PEV2 and PEV3 and examined their expression. The fluorescence patterns in yeast cells transformed with PEV2 and PEV3 expression vectors differed from that of EGFP expression alone, indicating that these fusion proteins were successfully expressed (Figure 9). However, since the expression levels of these fusion proteins were lower than those of GFP alone, they could not be detected by western analysis using anti-GFP antibodies. Thus, the expression of the fusion proteins was validated by northern blot analysis for the fused transcripts, which corresponded to the fluorescence levels. This is the first report to visualize the intracellular expression of endornaviral proteins and may lead to further studies on the genetics of endornaviruses by using yeast heterologous expression systems.

Previously, we reported that the heterologous expression of ORF4 of Magnaporthe oryzae chrysovirus 1-A and ORF2 of *Alternaria alternata* chrysovirus 1, both belonging to the genus *β-chrysovirus*, suppressed yeast cell growth (Urayama et al., 2016; Okada et al., 2018). In the yeast heterologous expression of PEV2 and PEV3, growth retardation occurred during the logarithmic growth phases, but no reduction in viable cell number nor cell enlargement were seen (Figures 7, 8). Although the mycelial elongation rates of the host *Phytophthora* sp. infected with PEV2 and PEV3 were reduced, zoosporangium formation was significantly promoted (Uchida et al., 2021). In addition, the difficulty in obtaining virus-free strains suggests that PEV2 and PEV3 are maintained fairly stably in host cells. These results may also be related to the fact that host plants infected by many endornaviruses are symptomless.

In general, reverse genetics systems and *in vitro* transfection methods are used to study the effects of viral infection on host cells and the characteristic activities of virus-encoded proteins (Xu et al., 2012). These techniques are commonly used for animal and plant viruses, but have only been established for a few mycovirus species, including Cryphonectria hypovirus 1 (Choi and Nuss, 1992), Sclerotinia sclerotiorum hypovirus 2 (Marzano et al., 2016), Sclerotinia sclerotiorum ourmia-like virus 4 (SsOLV4) (Wang et al., 2020), Diaporthe RNA virus 1 (Moleleki et al., 2003), and Botrytis virus F (Córdoba et al., 2022). Well-known yeast RNA virus replication studies using cDNA cloning include *Saccharomyces cerevisiae* L-A virus (ScLAV), Saccharomyces 23S RNA narnavirus (ScNV-23S) (Esteban and Fujimura, 2003), and Saccharomyces 20S RNA narnavirus (ScNV-20S) (Esteban et al., 2005). In addition, cDNA of the plant viruses, Bromo mosaic virus (Ishikawa et al., 1997) and tomato bushy stunt virus (Panavas and Nagy, 2003), were expressed in yeast shuttle vectors, which led to the identification of yeast cofactor proteins in the replication of these plant RNA viruses. Thus, yeast-based research systems are expected to provide insights into virus-host interactions that cannot be obtained from the biological information of the original hosts alone.

Our results gave insights into the mechanism of nick generation in *Phytophthora* sp. Endornaviruses; we showed that the nick could arise as a dsRNA break structure and as a ssRNA transcript. This state is suggested by the reproducibility of nick generation in PEV3 expressed heterologously in yeast cells. The negative strand of the nick generated in yeast cells has not been detected so far, and it could not be detected by northern blot analysis after purification of dsRNA (data not shown). Therefore, nick fragments are expected to not exist as dsRNA, at least in yeast cells.

The weak effects of endornaviruses, which have persistently infected many species as hosts, have made it difficult to understand their potential intrinsic nature. In addition, it has been challenging to study endornaviruses at the cellular level because it has not been possible to establish an infection system by mechanical inoculation. Our system for heterologous expression of endornaviruses in yeast cells is expected to be a new research method that can address the above-mentioned issues. In this study, we focused on the existence and characterization of PEV2 and PEV3 nicks, and we report here the successful attempts to construct full-length cDNA clones and heterologous expression systems using yeast cells.

This report will accelerate our understanding and further our knowledge of endornaviruses, many of which are still uncharacterized, but found in many crops, and will broadcast their new-found value as valuable genetic resources in the field of agricultural production.

## Data availability statement

The original contributions presented in the study are included in the article/Supplementary material, further inquiries can be directed to the corresponding author.

## Author contributions

KS performed the experiments with academic and technical assistance from KK, TF, RO, and HM. KS, KK, and HM analyzed the data and wrote the first draft of the manuscript. All authors critically reviewed the manuscript and approved the final submission.

## Funding

This work was supported by a Grant-in-Aid for Challenging Exploratory Research from the Japan Society for the Promotion of Science (20KK0137) to HM.

## Conflict of interest

The authors declare that the research was conducted in the absence of any commercial or financial relationships that could be construed as a potential conflict of interest.

## Publisher’s note

All claims expressed in this article are solely those of the authors and do not necessarily represent those of their affiliated organizations, or those of the publisher, the editors and the reviewers. Any product that may be evaluated in this article, or claim that may be made by its manufacturer, is not guaranteed or endorsed by the publisher.
