# Supplementary material for: Successful full-length genomic cloning and characterization of site-specific nick structures of Phytophthora endornaviruses 2 and 3 in yeast, Saccharomyces cerevisiae
Source: Front Microbiol. 2023 Sep 12;14:1243068. doi: 10.3389/fmicb.2023.1243068 (PMC10523305; doi:10.3389/fmicb.2023.1243068)
Supplement: Supplementary file 1 [file Data_Sheet_1.docx]

Supplementary Material

**Supplemental Table S1. List of the primers used in this study.**

**
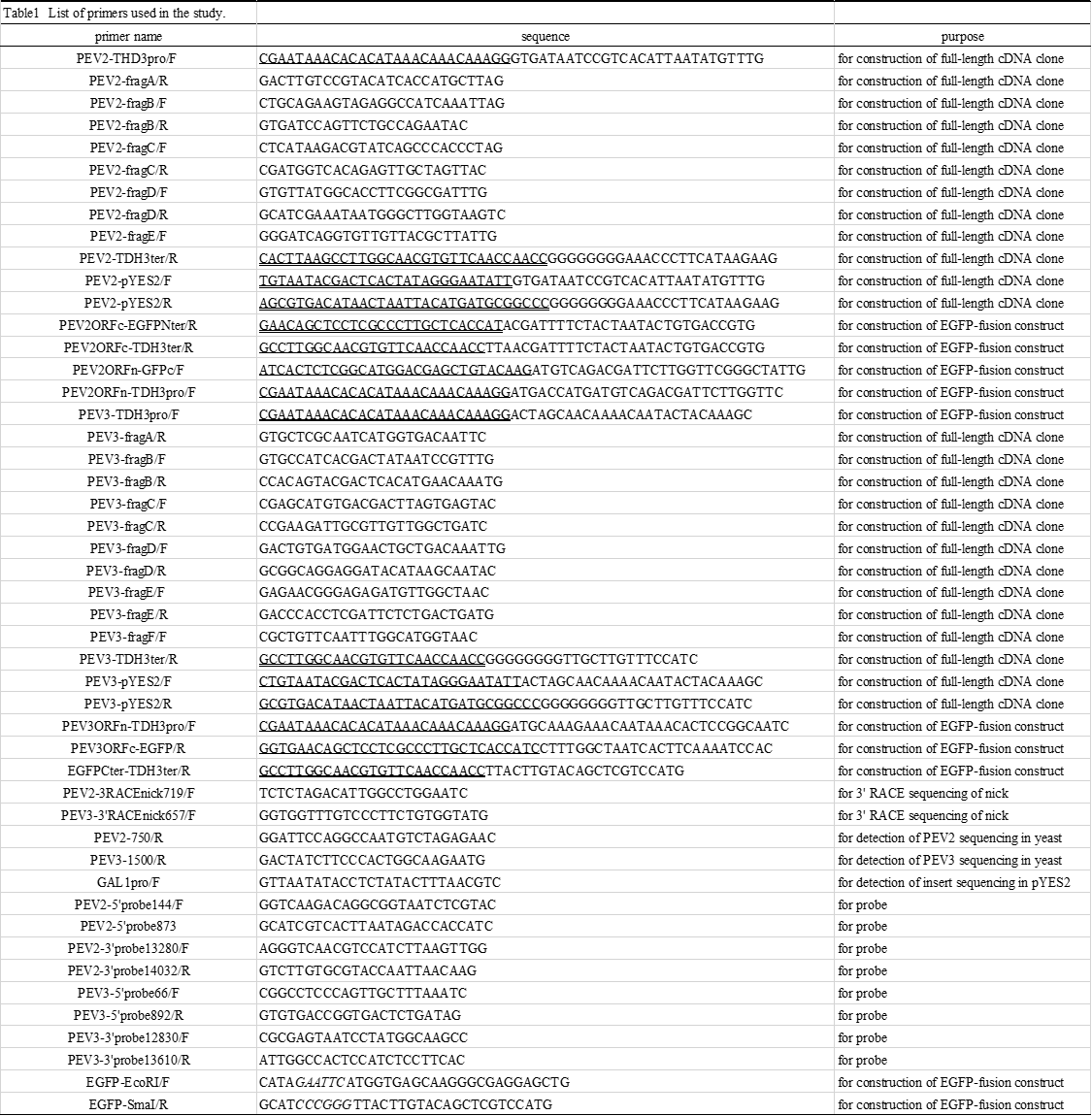
**

Underlined sequence is homologous to other primer to facilitate homologous recombination in yeast. The overlapped restriction site is in italics.

**Supplemental Figures**

**
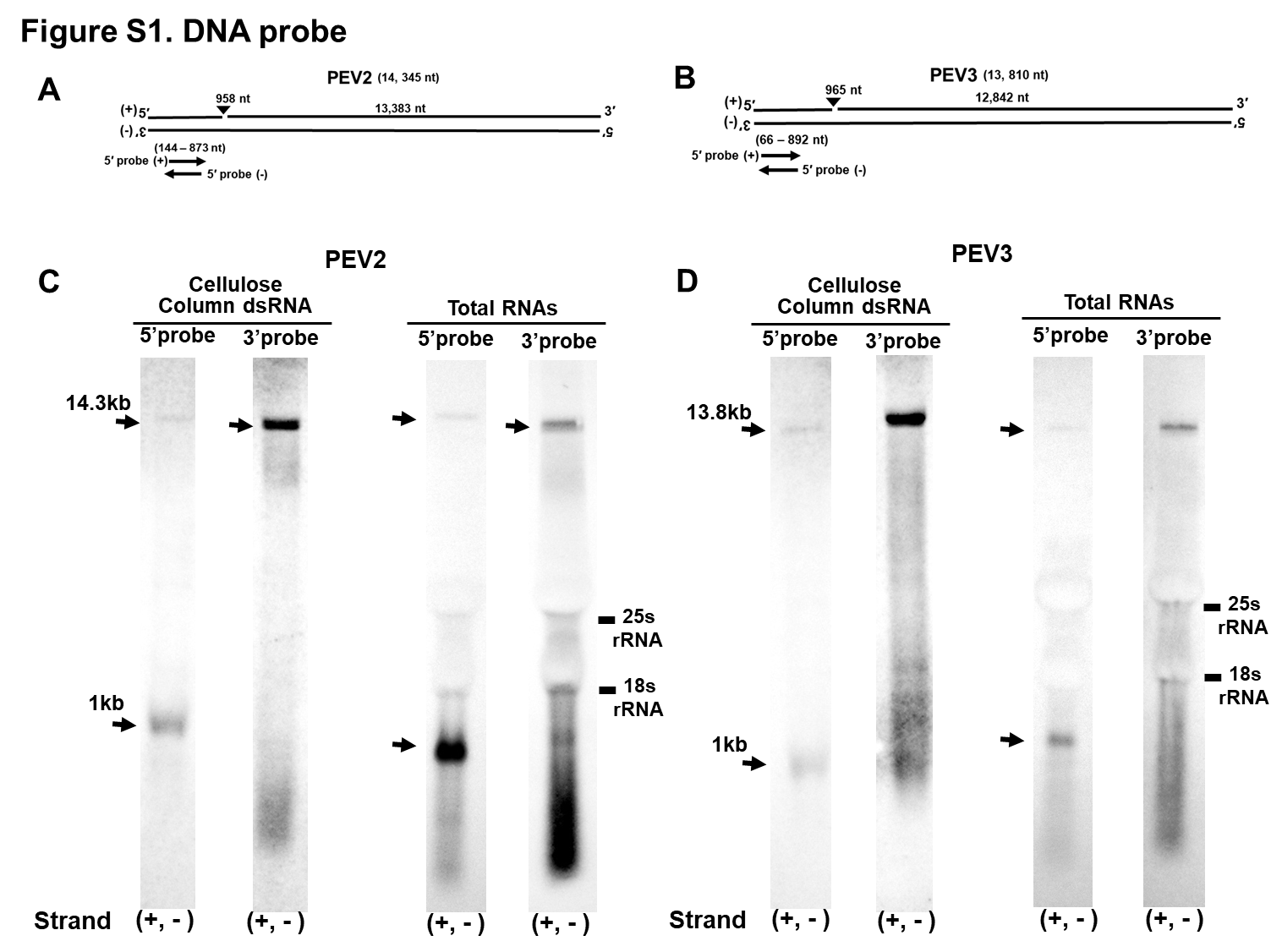
**

Northern blot hybridization detection of dsRNA extracted from strain CH98ASP059, and PEV2 or PEV3 genomic RNA and nick fragments in total RNA. 250 ng of dsRNA and 20 μg of total RNA were heat-denatured and applied. DIG-labeled DNA probes were used for detection, so that the positive and negative strands were detected simultaneously. (A) Whole genome of PEV2 and the position of DNA probes used. (B) Whole genome diagram of PEV3 and the position of DNA probes used. (C) Northern blot analysis of PEV3 using PEV2-5' and PEV2-3' probes. A 14.3 kb band indicating genomic RNA was detected with both probes. A 1 kb band indicating nick was detected only with the PEV2-5' probe. (D) Northern blot analysis of PEV3 using PEV3-5' and PEV3-3' probes. A 13.8 kb band indicating genomic RNA was detected with both probes. A 1 kb band indicating nick was detected only with the PEV3-5' probe. In the case of total RNA, 25S rRNA and 18S rRNA were detected nonspecifically.


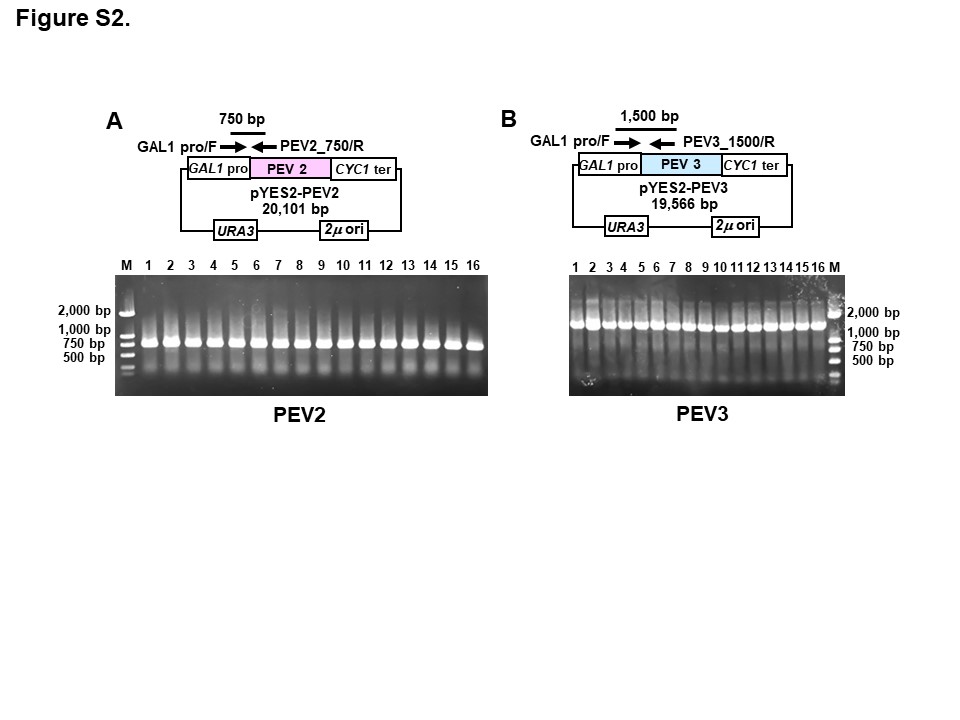


After transformation according to Figure 5, 16 single colonies were picked from SC-uracil agar plates and total nucleic acids were extracted. Using total nucleic acids as template, PCR was performed using primers specifically amplifying PEV2 or PEV3 (PEV2_750/R, PEV3_1500/R) in combination with primers specifically amplifying *GAL1* promoter (*GAL1* pro/F). (A) pYES2-PEV2 (*GAL1* promoter) construct, location of primer sets. Amplified products were confirmed by 1.0% agarose gel electrophoresis. M: DNA marker; 1~16: PCR products amplified from pYES2-PEV2 transformants (750 bp). (B) pYES2-PEV3 (*GAL1* promoter) construct, primer set locations. Confirmation of amplified products by 1.0% agarose gel electrophoresis. M: DNA marker; 1~16: PCR products amplified from pYES2-PEV3 transformants (1500 bp).


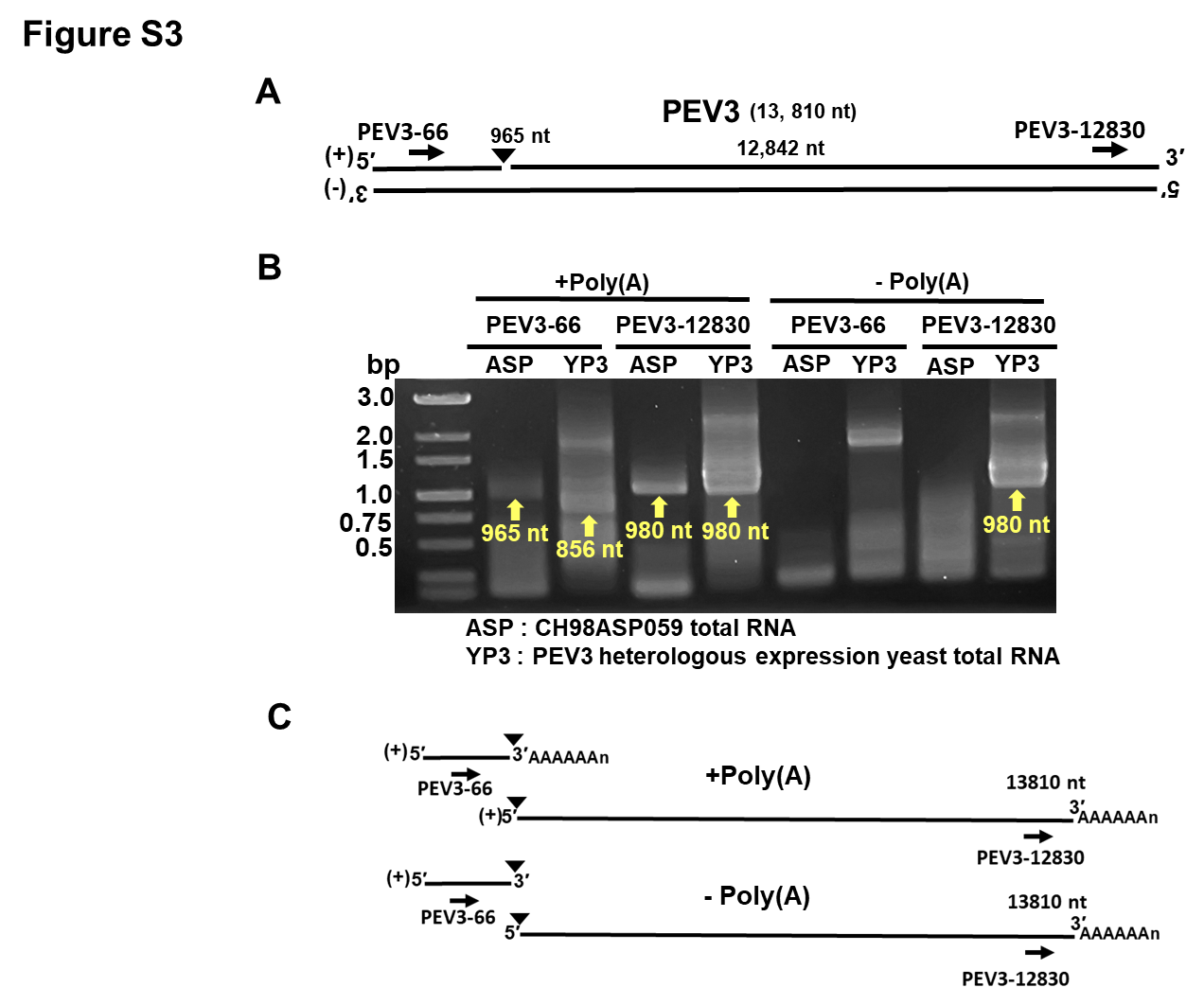


Determination of the sizes of the nick fragments occurring in PEV3 heterologous expression yeast (YP3) or in the Phytophthora strain CH98ASP (ASP) to confirm the presence of poly(A) tail by the 3' RACE method. We used 20 μg of total RNA extracts from the YP3 cells and 20 μg of total RNA extracted from the ASP mycelia. (A) Whole genome diagram of PEV3 and locations of the two primers, PEV3-66 (for Nick) and PEV3-12830 (for 3' terminus) for 3' RACE. (B) Agarose gel (1%) electrophoresis (50V, 75 min) of the 3' RACE amplified with PEV3-66 and oligo d(T)_18_ primers or PEV3-12830 and oligo d(T)_18_ to confirm the presence of poly(A) tails at the 3' ends of the ssRNA(+) transcripts. +Poly(A): total RNA was polyadenylated by poly(A) polymerase before RT-PCR. - Poly(A): without the poly(A) tailing reactions. (C) Schematic diagrams of PEV3 genome in PEV3 heterologous expression yeast. +Poly(A): total RNA was polyadenylated by poly(A) polymerase before RT-PCR. - Poly(A): without the poly(A) tailing reactions.
